# Members of the Lactococcus–Pseudolactococcus clade exhibit extensive genomic diversity in features associated with phage–host co-adaptation

**DOI:** 10.1099/mgen.0.001863

**Published:** 2026-10-12

**Keywords:** anti-phage, cell surface receptor, dairy, lactic acid bacteria, polysaccharide, prophage

## Abstract

*Lactococcus lactis* and *Lactococcus cremoris* are widely exploited in global dairy fermentations. Consequently, strains of these species are extensively studied, as are their interactions with their infecting bacteriophages. In recent years, the number of lactococcal species has increased significantly and the ecology of these species extends far beyond the dairy niche. Furthermore, this expansion has also warranted the reassignment of several former lactococcal species to the genus *Pseudolactococcus*. In the present study, we explored the bacteriophage-relevant features of 495 genomes within the *Lactococcus–Pseudolactococcus* clade, including regions associated with (1) cell wall polysaccharides (CWPS) and exopolysaccharides (EPS) as possible bacteriophage receptors, (2) prophages and phage satellites and (3) anti-phage systems. This analysis revealed the universal presence and consistent genomic location of *cwps* loci, with extensive relationships between the loci of distinct species of both genera. It was also established that EPS-associated gene clusters are present in approximately half of the evaluated species and occur in a strain-dependent manner, suggesting that they are associated with niche adaptation rather than a core biological function of these organisms. Significant diversity and phylogenetic overlap of prophages across several species of both genera were observed. While prophages were widespread, phage satellites were especially prevalent among dairy-associated species. At the strain level, prophage, satellite and defence-system abundances were positively correlated. Restriction–modification systems appeared to form the core defence strategy of the *Lactococcus–Pseudolactococcus* taxon, complemented by species- and strain-level tailoring of the anti-phage repertoire to respond to phage pressure in diverse ecological niches. Niche-associated differences observed within *L. lactis* underscored that greater apparent phage exposure was not uniformly reflected across all phage-relevant features: dairy-associated strains carried more prophages, satellites and defence systems than plant-associated strains, whereas plant isolates exhibited greater rarefied polysaccharide pellicle richness. Overall, this represents the most comprehensive genome-based analysis of members of the *Lactococcus–Pseudolactococcus* clade, guiding our current understanding of the evolutionary pathways underlying phage–host co-adaptation followed by these micro-organisms.

**Impact Statement**

To date, knowledge pertaining to the genetic diversity, functionality and interactions with bacteriophages in lactococci is largely confined to dairy-associated species of *Lactococcus* and *Pseudolactococcus*. However, the relationships of these species with other lactococcal and pseudolactococcal species and their evolutionary pathways have not been interrogated. Bacteriophages of *Lactococcus lactis* and *Lactococcus cremoris* are known to recognize saccharidic receptors and this prompted a detailed genome analysis of all members of this clade for which sequence information is available. This allowed significant insights into inter-species and inter-genus relationships of their bacteriophage-relevant features including cell wall polysaccharides, prophages, phage satellites and anti-phage strategies. This will guide our understanding of the evolutionary pathway of these organisms and provide evolutionary context for dairy-associated species, which appear to have undergone significant genome decay in their adaptation to the dairy niche.

## Data Summary

The authors confirm all supporting data and protocols have been provided within the article or through supplementary data files. The custom python script mentioned in section 6.1 can be found in Figshare [1].

## Introduction

The cell surfaces of Gram-positive ovoid bacteria contain diverse glycan structures that serve multiple functions in cell division, cell wall integrity maintenance and interactions with biotic and abiotic environmental factors [2, 3]. Among the Gram-positive lactic acid bacteria, the rhamnose-containing cell wall polysaccharides (CWPS) and exopolysaccharides (EPS) are also implicated as receptors for bacteriophages for several genera of lactococcal and dairy streptococcal phages [4–8]. Given the economic significance of dairy-associated species such as *Lactococcus lactis, Lactococcus cremoris* and *Streptococcus thermophilus,* it is perhaps unsurprising that the genetic regions associated with their synthesis of these saccharidic structures, especially CWPS, have received intense research attention [9–11].

The taxonomic and ecological diversity of lactococci has expanded substantially in recent years. Until relatively recently, mesophilic mixed starter cultures were assumed to be composed of strains of *L. lactis* and/or *L. cremoris* with *Leuconostoc* spp. in some cases. However, subsequent reports established that strains of *Pseudolactococcus raffinolactis* and *Pseudolactococcus laudensis* may also be members of these consortia, albeit at (substantially) lower relative abundance [12, 13]. In parallel, several new lactococcal species have been isolated from insect, plant, animal and dairy niches, adding further complexity to our understanding of the ecology, evolution and adaptation of lactococci [12, 14–23]. Phylogenomic analysis resolved two major lineages within this expanded taxon and supported the reclassification of several former *Lactococcus* species within the genus *Pseudolactococcus* [24]. The clade currently comprises 11 *Pseudolactococcus* species (*Pseudolactococcus carnosus, Pseudolactococcus chungangensis, P. laudensis, Pseudolactococcus paracarnosus, Pseudolactococcus piscium, P. raffinolactis, Pseudolactococcus hodotermopsidis, Pseudolactococcus plantarum, Pseudolactococcus insecticola, Pseudolactococcus reticulitermitis* and *Pseudolactococcus yaeyamensis*) and 15 *Lactococcus* species (*L. cremoris, L. lactis, Lactococcus hircilactis, Lactococcus allomyrinae, Lactococcus protaetiae, Lactococcus nasutitermitis, Lactococcus termiticola, Lactococcus muris, Lactococcus ileimucosae, Lactococcus garvieae, Lactococcus petauri, Lactococcus kimchii, Lactococcus taiwanensis, Lactococcus fujiensis* and *Lactococcus formosensis*). Since most of these emerging species have been reported over the past 5 years, their conserved or lineage-specific genomic features underlying their evolutionary diversification and adaptation to bacteriophages and various ecological niches remain poorly understood.

The CWPS biosynthesis loci of *L. lactis* and *L. cremoris* have been analysed in detail and four major *cwps* genotypes named A–D have been identified, with 11 *cwps* C subtypes also established [9, 25]. In these lactococci, the *cwps* locus encodes two distinct but functionally linked structures: a peptidoglycan-embedded rhamnan (synthesized by genes at the 5′ end of the *cwps* locus) and a surface-exposed side-chain structure or polysaccharide pellicle (PSP) synthesized by genes at its 3′ end [9]. Recently, we have identified that *cwps* loci are present in the genomes of *P. laudensis* and *P. raffinolactis* and comparative analysis revealed eight novel *cwps* genotypes (i.e. *cwps* E-L), highlighting the genetic diversity of these loci with some notable interspecies relationships also identified [26]. For example, the *cwps* clusters of *P. raffinolactis* Lr_18_12S (*cwps* K genotype) and *L. lactis* UC509.9 (*cwps* genotype A) were shown to exhibit significant similarity in organization and gene content, especially in the rhamnan-encoding region. In contrast, isolates assigned to genotypes E and G encompass *cwps* loci associated with production of a rhamnan-like polysaccharide, yet lacking an apparent PSP module [26]. It remains unknown whether *cwps* loci are universally distributed across the wider *Lactococcus–Pseudolactococcus* clade and, where present, whether their genetic organization and core features mirror the known architectures of dairy-associated species.

EPS represent an additional source of cell-surface variation. Beyond their valuable technological properties in food [27], EPS may influence phage susceptibility by masking cell-surface receptors or, in some cases, directly mediating phage adsorption [8, 28]. The associated *eps* loci for their biosynthesis has been established as being plasmid-derived in many lactococcal strains [8, 29, 30], although a small number of chromosomally encoded loci have also been reported [31]. In contrast, when present in *P. laudensis* and *P. raffinolactis, eps* loci were all observed to be located on their chromosomes, encompassed within apparent integrative and conjugative elements (ICEs) [26]. Variation in *cwps* and *eps* loci therefore generates a heterogeneous cell-surface landscape with the potential to shape phage adsorption, host range and resistance across lactococcal and pseudolactococcal lineages.

Following lactococcal phage adsorption to suitable host-encoded (typically saccharidic) receptors, injection of the phage DNA into the host cell cytoplasm may elicit a response by the host to limit the progression of phage replication. In *L. lactis* and *L. cremoris*, Restriction–modification (R–M) and abortive infection (Abi) systems are the most dominantly reported defence strategies [32, 33], while CRISPR-Cas systems have been reported to be present (although not functionally validated) in the genomes of *P. raffinolactis, P. laudensis* and other pseudolactococcal species [34, 35]. Phage satellites, mobile genetic elements which depend on helper phages for mobilization, provide an additional, non-canonical layer of defence. Among them, phage-inducible chromosomal islands (PICIs) hijack phage assembly and packaging functions to promote their own transfer, frequently at the expense of helper-phage reproduction, and can thereby provide population-level protection against infection [36]. Although knowledge of phage–host interactions and the phage defence landscape of the majority of lactococcal and pseudolactococcal species is limited, the presence of satellites and defence elements is a strong indicator that species encounter phages in their respective ecological niches. This, together with the presence of prophages in their genomes, complemented by the variation in loci encoding saccharidic surface receptors, may be used as a proxy to establish the presence and possible selective pressure that lytic and/or temperate phages may impose on strains of these species.

The variable presence of *eps* loci, combined with the omnipresence and observed diversity of the *cwps* loci in the four aforementioned dairy-associated species, prompted an analysis of the reference genomes of all described species for which genome sequence data were available, followed by detailed analysis of all available sequenced genomes for all species (October 2025, 495 genomes in total). In the current analysis, we evaluated (a) the presence and (b) diversity of *cwps* and *eps* loci and (c) their coincidence with prophages, phage satellites and host defence systems. This ‘phage-centric’ analysis approach across all known members of the clade will create significant insights into the common or niche-specific cell surface saccharidome and sheds light on the evolutionary pathways and co-adaptation with phages of members of the *Lactococcus–Pseudolactococcus* clade.

## Methods

### Cell wall- and exo-polysaccharide biosynthetic gene cluster identification and analysis

The annotated genome sequences of 14 reference lactococcal species and 11 pseudolactococcal species were downloaded from the NCBI genome database website (https://www.ncbi.nlm.nih.gov/datasets/genome/). The reference genome for each species identified within the NCBI genome datasets database, where available, is indicated in Table S1, available in the online Supplementary Material. If a complete reference genome was not available, the genome with the lowest number of contigs was selected as a representative of that species (Table S1). No genome sequence data was available for *L. kimchii*.

Artemis 18.2.0 [37] was used to visualize the genomes and to search for rhamnose-containing *cwps* gene clusters. This was performed by manually searching for the thymidylyltransferase *rmlA* gene that is conserved amongst several species [9]. Once identified, the surrounding genes were analysed to establish the presence and boundaries of the putative *cwps* loci. For *eps* locus identification, a similar approach was employed but ‘tyrosine kinase’ was the search term used to identify potential loci followed by manual interrogation of the surrounding gene content. A custom python script was developed using BioPython 1.85 to extract the locus tags of all the genes present in the *cwps* and *eps* clusters [1]. The extracted *cwps* and *eps* cluster sequences of each species were compared using Clinker with default settings [38] via the CAGECAT webserver, release 1.0 [39]. Subsequently, annotations of each gene present within individual gene clusters for each examined species were compared to that of *L. cremoris* NZ9000 (*cwps* locus) and *L. lactis* Tempeh6L (*eps* locus), respectively, for which the functional annotations of gene products have been evaluated in detail [40]. In cases where the gene product function could not be related directly to the reference strain, HHPred (October 2025) [41] and DeepTMHMM v1.0 modelling [42] were used to enhance the functional prediction and annotation.

## Phylogenetic analysis of *cwps* rhamnan-associated gene products

Artemis V18.2.0 [37] was used to extract the aa sequences of the proteins encoded by genes *rmlA-D* and *rgpA-D* within the rhamnan biosynthesis-associated region of the *cwps* loci of all available reference genomes. The files were concatenated and combined into a single file with an awk script in bash (see supplementary materials). clustalw V2.0 was used to create a multiple alignment of the sequences with default settings [43]. The number of bootstrap trials used was one thousand. The resulting unrooted trees were customized using ITOL v7.2.1 [44].

### Inter-species comparative genome analysis of *cwps/eps* gene clusters

The genome sequence of all available complete genome sequences or scaffolds of lactococcal and pseudolactococcal strains were downloaded from the NCBI database using ncbi-genome-download v0.3.3 [45]. This resulted in a database of 238 complete genomes and 257 scaffolds being generated. All genomes were reoriented to commence with *dnaA* using dnaapler v1.2.0 [46] and genomes were annotated with Bakta [47].

In *L. lactis* and *L. cremoris* genomes, *cwps* loci were identified using a custom Python script (see Data Summary) that parsed Bakta annotation TSV files with strand awareness to delineate the rhamnan- and PSP-encoding modules. For each genome, coding sequences (CDS) were sorted by genomic coordinate and the *cwps* region was anchored using *rmlA* (annotated as *rfbA*). A PSP marker was then identified within ±120 ORFs of *rfbA* as a gene annotated as either a ‘DUF2304 domain-containing protein’ or an ‘uncharacterized conserved protein’, corresponding to the conserved gene *wpsB*. Locus boundaries were defined using strand-specific rules. On the positive strand, the rhamnan segment was defined from the start of *rfbA* to the stop codon of the gene located two ORFs upstream of the PSP marker [corresponding to the gene upstream to the *wpsA* priming glycosyltransferase (GT)]; the PSP segment was defined from the start of the gene immediately upstream of the marker (*wpsA*) to the last consecutive same-strand ORF downstream of the marker, excluding opposite-strand insertion sequence (IS) elements/transposases/integrases. On the negative strand, the rhamnan segment was defined from the start codon of the gene located two ORFs downstream of the PSP marker to the stop codon of *rfbA*; the PSP segment was defined from the uppermost same-strand CDS as the PSP marker excluding opposite-strand IS elements/transposases/integrases, to the first same-strand ORF immediately downstream of the PSP marker. For other *Lactococcus* and *Pseudolactococcus* genomes, the same script was utilized to detect candidate *cwps* loci, followed by manual curation to confirm rhamnan and PSP-encoding region boundaries.

Candidate *eps* loci were identified by querying Bakta annotations for the keywords ‘cpsC,’ ‘cpsD,’ ‘epsD,’ ‘cell envelope’ and ‘LytR.’ Genomes obtaining positive hits were manually inspected to ascertain the presence of a true *eps* cluster based on the presence of genes encoding hallmark functions like the Wzy polymerase and the Wzx flippase and to establish its boundaries, which typically spanned from *cpsC* to a gene annotated as a ‘cell envelope transcription attenuator’ (corresponding to the OrfY modulator). In scaffold assemblies, *eps* loci located at contig termini were classified as intact only and used for downstream analyses if the region extended through *orfY*.

Once identified, the nt sequences of the regions that encode the *cwps* and *eps* loci were extracted using samtools v1.22.1 [48] and re-annotated with Bakta v1.9.4 [47], utilizing the v5.1 full database. Pangenome analysis was performed with Roary v3.13.3 [49] separately for the *cwps* (including the full locus, as well as the rhamnan- and PSP-encoding regions independently) and *eps* loci at blastp identity thresholds of 30, 50 and 70%. The resulting gene family presence/absence files were manually interrogated and modified to correctly assign the cluster-specific functions to each gene based on homology to previously established clusters of related dairy-associated species including *Lactococcus* spp., *Pseudolactococcus* spp. and *S. thermophilus* [10, 26, 40]. Where sequence homology was deemed insufficient to warrant functional annotation, the gene products were analysed using transmembrane topology prediction in DeepTMHMM v1.0 [50], HHPred [41] and the Conserved Domain Database v3.21 [51]. Visualization of the loci comparisons was generated using Clinker through the CAGECAT webserver, release 1.0 [52]. Heatmaps were generated using a custom python script. Horizontal gene transfer (HGT) origins of chromosomally encoded *eps* loci were evaluated by manual inspection of the gene annotation files, as well as using Alienhunter v1.7 [53] and mobileOG-db v1.1.3 [54] via the Proksee webserver [55], IslandViewer4 (October 2025) [56], OriTdb2 [57] and ICEfinder from the ICEberg 3.0 webserver [58], as needed.

### Detection of anti-phage systems, prophages and phage satellites

Phage defence systems were predicted utilizing PADLOC v2.0.0 [59] for the entire dataset, whereas prophages, phage satellites and CRISPR-Cas systems were predicted from the set of complete genomes (*n*=238). Prophages were predicted with geNomad v1.8.1 [60] in conservative mode. Predicted sequences were evaluated for completeness and contamination utilizing CheckV v1.0.1 [61]. Host-free sequences classified as either complete, high-quality (>90% completeness), as well as medium-quality sequences with >80% completeness, were subsequently annotated using Pharokka v1.7.3 [62]. Resulting gene annotations were manually inspected and only prophages encoding an apparently complete set of structural genes, including at least head, tail, packaging and integrase genes, were considered ‘intact’, while the remaining ones were considered ‘incomplete’. A proteome-based phylogenetic tree with random species representatives was generated using ViPtree v4.0 [63] and edited with iTOL v7.2.1 [44]. Phage satellites were predicted using SatelliteFinder v0.9.1 [64], implemented via MacSyFinder v2.1.1 [65]. Each strain was analysed independently against all four PICI family models (PICI, P4, PLE and cfPICI) using --db-type ordered_replicon. Lastly, CRISPR-Cas systems were predicted using CRISPRCasFinder 4.3.2 and the resulting high-confidence spacers were blast-searched (blastn 2.15.0+) against the 238 study genomes (≥95% identity/≥90% coverage) and against a 466-genome lactococcal phage database (INPHARED, 14 April 2025; ≥90% identity/≥90% coverage). Target elements, genes and phage isolation hosts were assigned from Bakta and GenBank annotations; with full parameters reported in Table S2.

## Results

### CWPS loci are highly diverse and universally present among lactococci

While the involvement of dairy lactococcal CWPSs in phage–host interactions has underpinned the detailed comparison and analysis of the *cwps* loci of dairy-associated species, the presence and diversity of these loci in other lactococcal and pseudolactococcal species has not been defined to date. In the present study, the reference genomes of each of 25 (of the 26 known) species for which genome sequence data was available were analysed for the presence of an identifiable *cwps* locus (genome sequence for a representative strain of *L. kimchii* is not currently available). All 25 (14 lactococcal and 11 pseudolactococcal) species were each observed to harbour a single *cwps* locus ranging in size from ~26 to 34 kb in lactococcal species and between 16 and 30 kb in pseudolactococcal species. Furthermore, the overall organization and genomic location of these gene clusters (relative to *dnaA*) is similar across all species (Fig. 1).

**Fig. 1.**
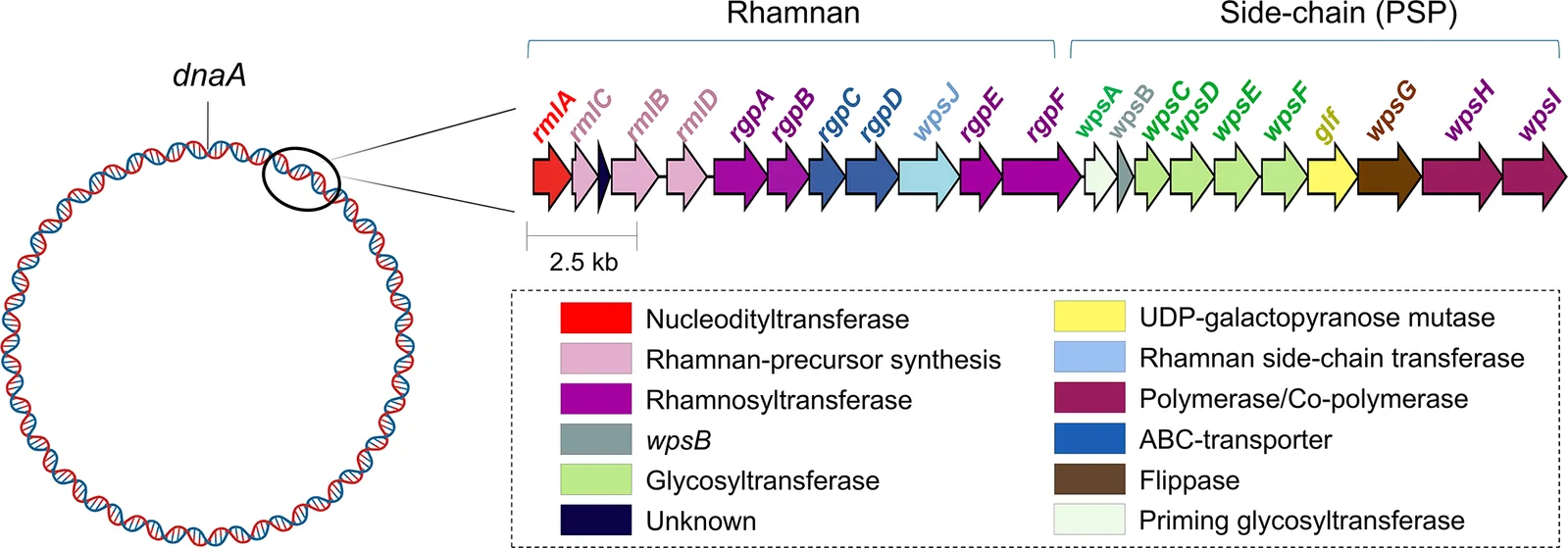
Schematic depicting the genomic position of the *cwps* locus (circled section) in lactococcal chromosomes relative to *dnaA* (left). The genomic position and gene organization are representative of all analysed species. An expanded view of a representative *cwps* locus highlighting the typical genes, their associated functions and their location within the rhamnan- or side-chain-associated region of the locus (right).

The *cwps* loci of dairy lactococci are established to encode the synthesis of two distinct and interconnected structures: a peptidoglycan-embedded rhamnan (encoded by the 5′ end of the *cwps* locus) and a surface-exposed side-chain structure (encoded by the 3′ end of the *cwps* locus) [40]. To determine the broader diversity of the *cwps* loci of lactococcal and pseudolactococcal species, the *cwps* loci of 495 strains for which the complete genomes (*n*=238) or genome scaffolds (*n*=257) were available, were analysed. Among the 495 loci analysed, 469 were observed to harbour an intact *cwps* gene cluster (26 *cwps* gene clusters appeared to be truncated in the scaffold assemblies analysed).

#### Rhamnan-encoding region of the *cwps* locus

The rhamnan biosynthesis-associated region of the lactococcal and pseudolactococcal *cwps* loci comprises the genes associated with rhamnose-nt precursor synthesis (*rmlA-D*) and rhamnan structure synthesis (*rgpA-B*) and transfer to the outer face of the cell membrane (*rgpC-D*) (Fig. 1). Comparative analysis of the rhamnan-associated biosynthetic gene products of the 469 intact *cwps* loci highlighted three major phylogenetic groups: (1) a group that incorporates 108 strains of *L. lactis*, *L. cremoris* and *L. hircilactis*, which are all dairy-associated species, while it also includes one *P. reticulitermitis* strain; (2) a group of 134 strains which exclusively belong to lactococcal species including *L. lactis*, *L. cremoris*, *L. taiwanensis*, *L. fujiensis* and *L. termiticola;* and (3) a group (*n*=227) that represent species of both *Lactococcus* and *Pseudolactococcus* including *L. lactis*, *L. cremoris*, *L. protaetiae*, *L. allomyrinae*, *L. garvieae, L. petauri, L. formosensis* and *L. muris* as well as *P. laudensis, P. raffinolactis, P. carnosus, P. paracarnosus, Pseudolactococcus homotermopsidis, P. plantarum* and *P. insecticola*. Interestingly, the pseudolactococcal rhamnan-encoding loci form a discrete phylogenetic group except for *P. reticulitermitis,* which aligns more closely within the dairy species in the first phylogenetic group.

In general, there is higher sequence similarity in genes located at the rhamnan biosynthesis-associated region of the *cwps* loci in all analysed reference strains, compared to those at the rightward (3′) end (side-chain or PSP biosynthesis-associated region), consistent with previous studies of the *cwps* of dairy lactococcal and pseudolactococcal species [5, 9, 26]. Nonetheless, pangenome clustering of the rhamnan module across 469 strains (using a threshold of 30% blastP identity which is assumed to capture the enzymes of similar function) revealed 95 distinct rhamnan biosynthesis-module genotypes based on their gene presence/absence, of which 54 were singletons (Fig. 2). To further capture closely related variants, we also grouped genotypes by single-linkage clustering of strains differing by ≤1 gene in the 30% identity-based presence/absence matrix, excluding transposases and hypothetical proteins, i.e. strains were assigned to the same group if they shared all or all but one genes with any other member of the group, except for genes encoding transposases or hypothetical proteins. This resulted in 44 inferred genotype groups with variable presence of one or more species (Fig. 3a). Interestingly, the rhamnan biosynthesis-encoding regions exhibit substantial sequence similarity across multiple *Lactococcus* species (e.g. between strains of *L. garvieae, L. petauri, L. formosensis, L. ileimucosae* and *L. muris*). Furthermore, there is at least one instance in *P. raffinolactis* in which this similarity extends across genera (Fig. 3a). However, it is obvious that without sequence data for additional isolates of the recently identified species of both genera, the true extent of the inter-species ‘sharing’ of these regions cannot be accurately predicted but likely extends beyond what is currently possible to define.

**Fig. 2.**
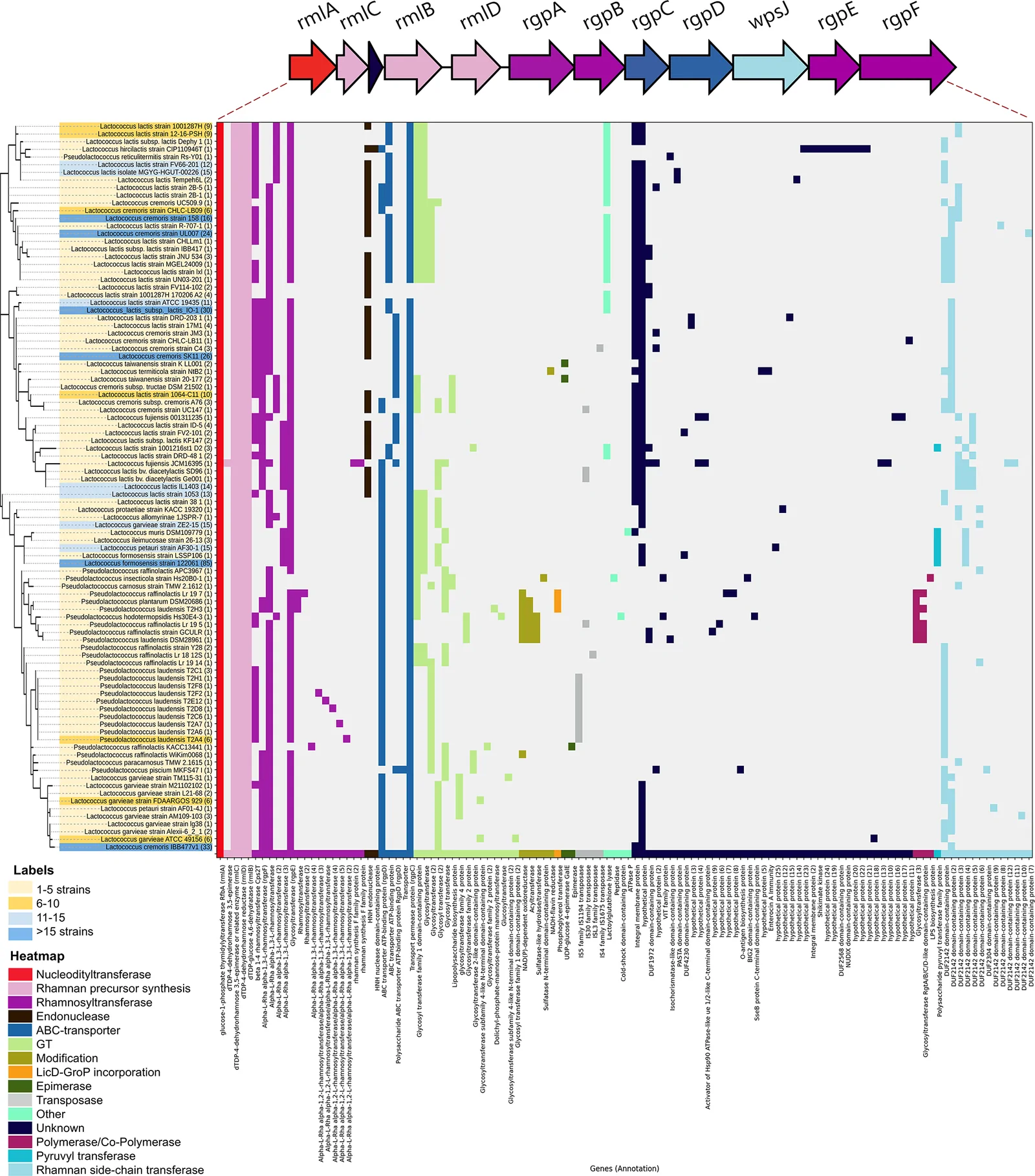
Gene presence/absence matrix for the rhamnan-encoding module of *cwps* loci across *Lactococcus* and *Pseudolactococcus* strains. Genes are grouped by predicted functional category. Presence/absence was determined from pangenome clustering using a blastP aa identity threshold of 30% to define homologous genes, accommodating substantial sequence divergence. The phylogeny is collapsed to representatives with distinct gene content profiles, with numbers in parentheses indicating how many strains each representative summarizes. The schematic at the top corresponds to the rhamnan-encoding region of the well-characterized *L. cremoris* NZ9000 and is provided as a reference for module content; total gene composition and synteny are not implied to be conserved across strains. Gene annotations are from Bakta, while annotations in parentheses correspond to the NZ9000 annotation.

**Fig. 3.**
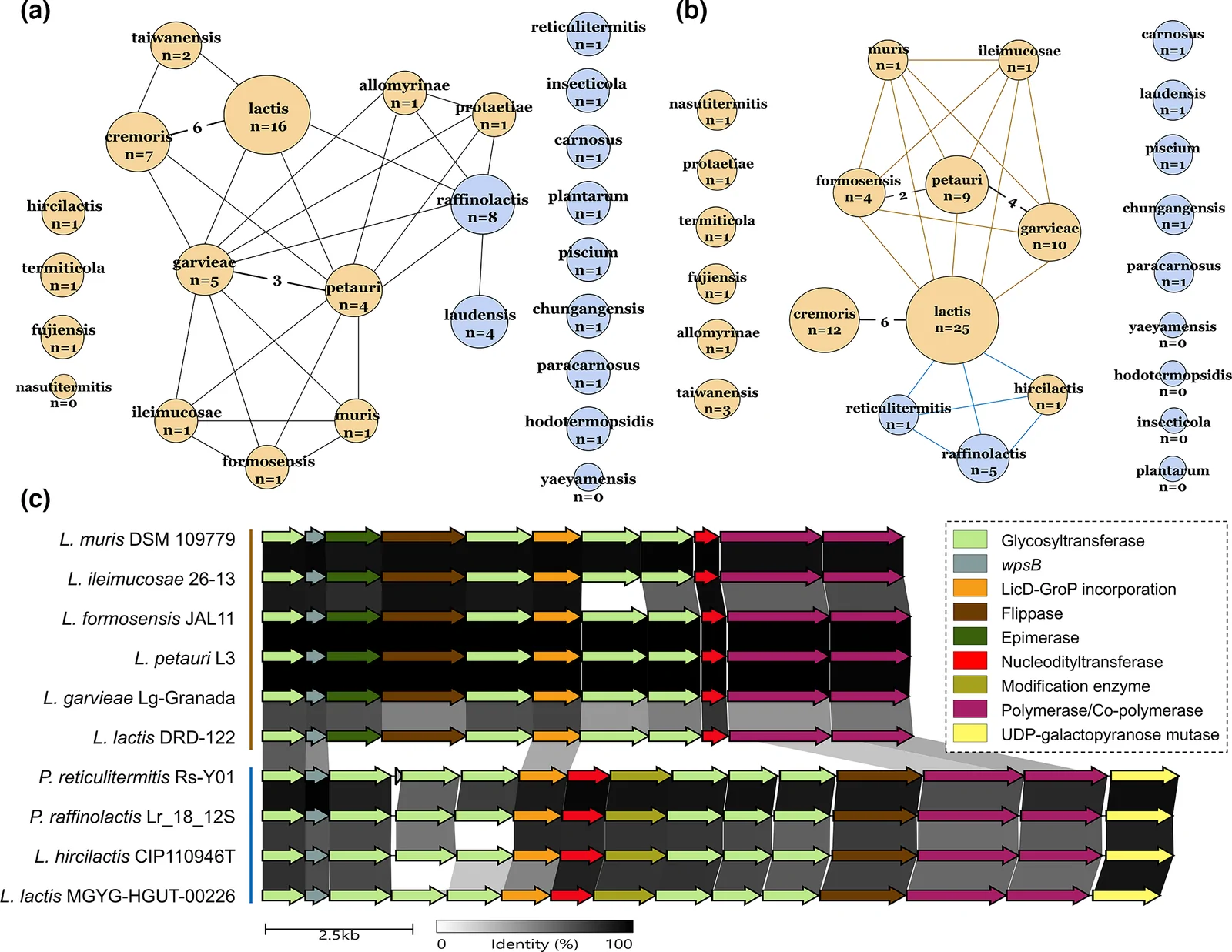
Relationship between the (**a**) rhamnan- and (**b**) PSP- (side-chain) encoding modules of *Lactococcus* (yellow) and *Pseudolactococcus* (blue) species. Groups were defined by single-linkage clustering of strains with ≤1 gene difference in their 30% homology gene presence/absence matrix, excluding transposases and hypothetical proteins (i.e. a strain was included in a group if it shared all or all-except-one genes with any other member of the group). Node sizes represent the number of groups (n) a species is included in. Linkages represent how many groups are shared between species, with lines containing no number representing a singleton. Coloured linkages in (**b**) are represented in panel (**c**), in a schematic depicting the side-chain-encoding regions of representative strains, highlighting the significant sequence homology of several genes within this region of the *cwps* locus between lactococcal and pseudolactococcal species. Colours associated with the strain name (indicated by a beige or blue line between the strain names and gene diagram for each strain) match with the colour of linkages joining the groups in panel (**b**).

In terms of gene content of the rhamnan biosynthesis-encoding modules, the rhamnose nt-biosynthesis genes *rmlA-D* were present and conserved across all strains of both *Lactococcus* and *Pseudolactococcus*. In contrast, the presence of the rhamnan structure synthesis rhamnosyltransferase (RT) encoding genes *rgpAB* and *rgpF* (whose gene products are associated with rhamnan side-chain elongation) was variable. GTs, including RTs, build the polysaccharide by sequentially adding sugar moieties. Pangenome clustering of predicted GT/RT proteins identified 31 distinct homologous gene clusters using a 30% blastP identity threshold (Fig. 3a) and 81 clusters using a 70% threshold, providing relaxed and stringent estimates of enzymatic diversity, respectively, within the rhamnan module. Among these, the RgpE RT was the most widespread, present in all 469 strains except *P. raffinolactis* KACC 13441 (=DSM 20443), *P. carnosus* TMW 2.1612, *P. insecticola* Hs20B0-1, and the *L. lactis* strains FV114-102, P83-3, 1001287 h_170206_A2, 1001287st2_A2, FV98-102 and_LZK-02. In *L. cremoris,* RgpE is suggested to be involved in rhamnan chain termination through the addition of a sugar at the non-reducing end of the rhamnan [40]. Interestingly, these six *L. lactis* strains were also the only genomes in the dataset lacking the ABC-type transporter typically associated with rhamnan repeat-unit export across the cytoplasmic membrane, suggesting an alternative export route or alternative structure types in these strains (see example of *L. lactis* LZK-02 in Fig. S1). It also suggests that additional non-canonical *cwps* genotypes or lineages exist.

Notably, eight *Pseudolactococcus* strains (*P. laudensis* DSM 28961 and T2H3, *P. raffinolactis* Lr_19_7, Lr_19_5 and GCULR *P. insecticola* Hs20B0-1, *P. plantarum* DSM 20686 and *P. hodotermodopsis* Hs30E4-3) did not encode a *wpsJ* gene, which encodes the rhamnan side-chain transferase that mediates the attachment of the PSP side chain to the rhamnan backbone in dairy lactococcal species [40]. Additionally, these strains did not encode key functional genes for PSP synthesis and export, including a flippase and the conserved WpsB protein, which is consistent with the lack of the WpsJ-encoding gene in these strains (Fig. S2). Four of these strains were previously noted [26] and chemical analysis of *P. laudensis* DSM 28961 confirmed the absence of a classical PSP. The absence of the above-mentioned genes in addition to the experimental validation of a representative strain lacking the PSP structure strongly suggests the absence of a canonical PSP in all eight *Pseudolactococcus* strains. Additionally, the presence of a NAD(P)-dependent oxidoreductase in all strains (except *P. insecticola*) suggests that the structure could be a talose-containing structure instead of a rhamnan, as has previously been experimentally demonstrated in *P. laudensis* DSM 28961 [26].

Finally, comparative analysis of the rhamnan-associated regions across all available strains from dairy and non-dairy species of lactococci and pseudolactococci has revealed that there is a significant number of individual genes in this region whose products are of unknown function. These genes likely contribute to strain-specific rhamnan structures and/or their modification (Fig. 2).

#### PSP/side-chain-encoding modules of the *cwps* locus

The side-chain-associated 3′ regions of the *cwps* clusters are highly diverse. Pangenome clustering at 30% cut-off blastP identity resulted in the identification of 85 predicted distinct PSP module genotypes, of which 47 were singletons (Fig. 3). Applying the same single-linkage grouping approach utilized for the rhamnan regions (≤1 gene difference; excluding transposases and hypothetical proteins) yielded 66 PSP groups, 22 more than observed for the rhamnan module (Fig. 3b).

Despite this overall diversity, we observed that the side-chain-encoding regions of the *cwps* locus of certain strains of distinct lactococcal species (i.e. strains from *L. muris, L. formosensis* and *L. ileimucosae*) were almost identical at the sequence level (Fig. 3b and c). Two of these strains were isolated from murine caecal (*L. muris*) and ileal (*L. ileimucosae*) samples, whereas the third is derived from plant material (*L. formosensis*), thus highlighting the overlap of these gene modules in species associated with distinct ecological niches. Similarly, the side-chain-encoding regions of certain strains from dairy-associated species including *L. hircilactis, L. lactis* and *P. raffinolactis* were observed to share significant sequence similarity with *P. reticulitermitis* strain Ra-Y01 (isolated from the gut of the Japanese termite, *Reticulitermes speratus*), highlighting inter-species and even inter-genus overlap of these loci (Fig. 3b and c).

PSP-group composition also varied with ecological niche. Within *L. lactis*, the most highly represented and ecologically diverse species in the dataset, plant-associated isolates exhibited greater rarefied PSP richness than milk/dairy isolates among strains with available niche metadata and assigned PSP groups (expected richness, 9.00 vs 5.73 at *n*=20; *P*=0.039). Other niches exhibited high observed richness relative to their sample sizes but were represented by ≤5 strains and were therefore not compared due to insufficient sample size. Notably, the greatest cross-species PSP-group overlap occurred between species occupying similar niches: *L. lactis* and *L. cremoris* shared six groups, whereas *L. garvieae* and *L. petauri* shared four groups. However, the respective contributions of ecological niche and shared ancestry to these patterns could not be unravelled.

The overall gene content of the side-chain module is characterized by a conserved priming GT, *wpsA* (except in *L. taiwanensis* K_LL001 and_K_LL004, *P. raffinolactis* Lr_19_14 and six strains of *L. lactis*) and a conserved membrane-associated accessory protein, *wpsB*, present among all studied PSP regions at the 5′ end of the locus (Fig. 4). Among the analysed reference lactococcal *cwps* loci, all but one (*L. fujiensis*) appears to possess polymerase/co-polymerase-encoding genes (*wpsH/I*) (Fig. S3), while these genes are not readily identifiable among the majority of pseudolactococcal species reference genomes analysed in this study. Upon examination of the full available dataset, 84 strains lacked polymerase/co-polymerase functions within their PSP loci, of which 70 were *L. lactis/cremoris* (Fig. 4). Similarly, a flippase-encoding gene, responsible for the transfer of the side-chain structure to the outer face of the cell membrane, appears to be universally present among both lactococcal and pseudolactococcal species, with the exception of *L. formosensis* RTP21489st1_C6 (Fig. 4).

**Fig. 4.**
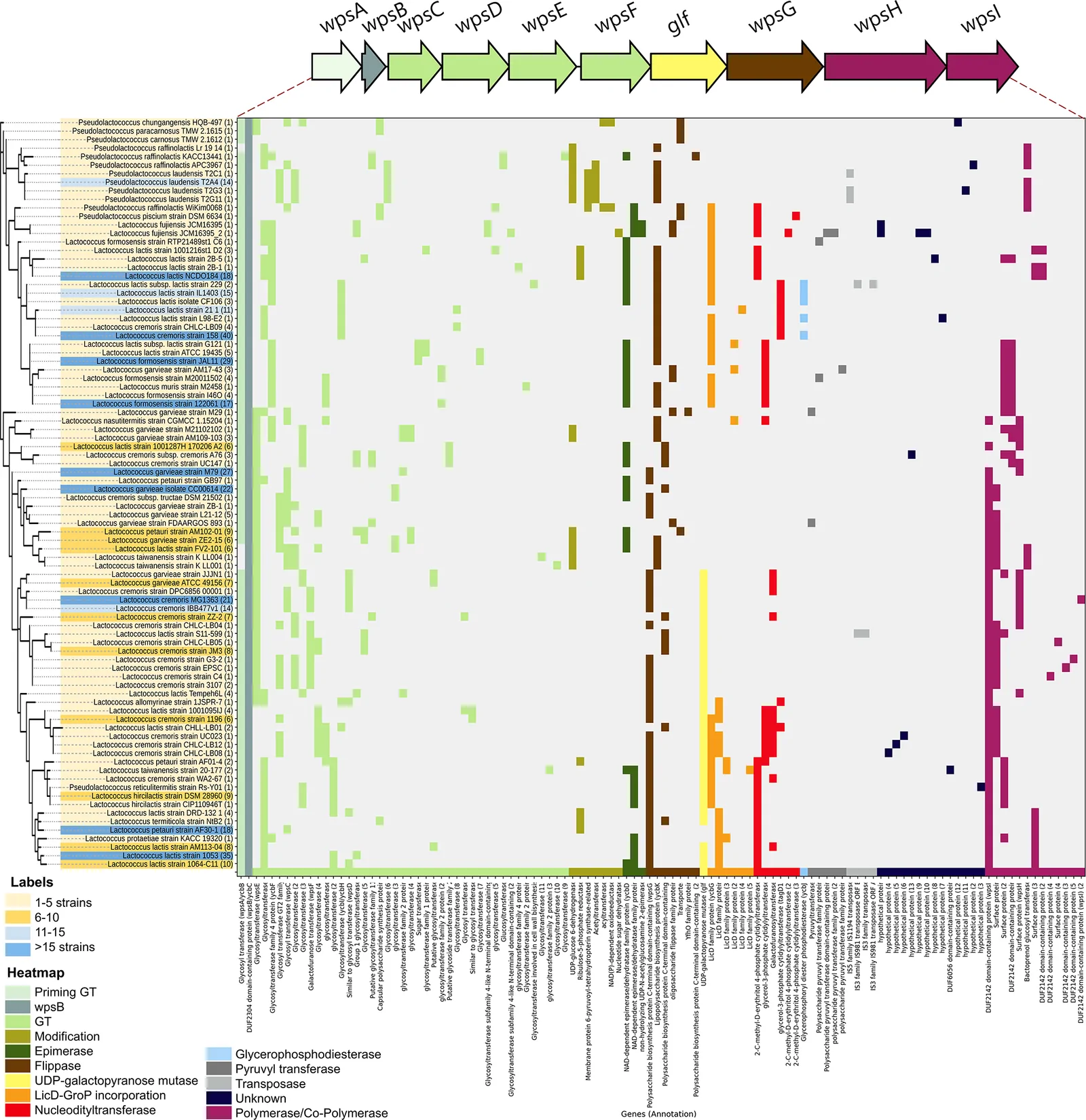
Gene presence/absence matrix for the PSP-encoding module of *cwps* loci across *Lactococcus* and *Pseudolactococcus* strains excluding the eight pseudolactococcal strains that lack the PSP-related gene content. Genes are grouped by predicted functional category. Presence/absence was determined from pangenome clustering using a blastP aa identity threshold of 30% to define homologous genes, accommodating substantial sequence divergence. The phylogeny is collapsed to representatives with distinct gene content profiles, with numbers in parentheses indicating how many strains each representative summarizes. The schematic at the top corresponds to the PSP-encoding region of the well-characterized *L. cremoris* NZ9000 and is provided as a reference for module content; total gene composition and synteny are not implied to be conserved across strains. Gene annotations were produced using Bakta, while annotations in parentheses correspond to the NZ9000 annotation based on functional studies.

The remainder of the genes were variable and highly diverse, including those encoding epimerases, mutases, pyruvyl transferases, modification enzymes (e.g. acetylases) and GTs, among others. Consistent with this variability, pangenome clustering of predicted GT/RT identified 42 distinct homologous gene clusters using a 30% blastP identity threshold (Fig. 4), increasing to a remarkable 137 at 70% blastP identity.

#### EPS-associated loci are variably present among lactococci and pseudolactococci

To explore if *eps* loci are similarly present in some or all species and to justify detailed analysis of all sequence data, an initial analysis of the reference genomes was undertaken. Among the 25 reference genomes analysed in this study, 9 species (*L. nasutitermitis, L. hircilactis, L. protaetiae, L. allomyrinae, P. paracarnosus, P. carnosus, P. laudensis, P. chungangensis, P. hodotermopsidis*) were confirmed to possess an *eps* cluster on their chromosomes (Fig. S4). The 5′ end of these loci typically harbours genes associated with regulation and chain length determination (chain length regulator, tyrosine kinase and phosphoesterase encoded by *epsCDB*), while the remainder of the gene cluster harbours a highly variable and strain-specific GT-encoding gene content (Fig. S4). It is possible that other species may harbour plasmid-encoded *eps* clusters as has been reported in *L. lactis/cremoris* [25, 66]. It is noteworthy that the EPS biosynthetic processes in lactococci are significantly less well studied in comparison to those of CWPS with the exception of seminal early studies [67, 68] and with no equivalent experimental validation of EPS biosynthetic pathways in the more recently described pseudolactococcal species.

Based on the observation that *eps* loci are present in multiple species, all available genome sequences were further interrogated for the presence of these loci. A total of 186 of the 495 analysed strains' genome sequences (85 associated with complete genomes; 101 associated with scaffolds) were observed to harbour an *eps* locus based on the presence of ‘*cpsCD*’ modulatory genes and hallmark genes such as the *wzx* (encoding a flippase) and the *wzy* (encoding a polymerase) genes, with some strains (*n*=5–6) harbouring two or more such loci. Among the strains harbouring two such clusters are *L. cremoris* JM4 which possesses a chromosomally and a plasmid-associated *eps* locus, while in *L. lactis* strains FM03 and S50 and in the *P. paracarnosus* strain two chromosome-borne *eps* clusters are present. The chromosomally encoded clusters of *L. lactis* FM03 and S50 are co-located and separated by a region rich in transposase-encoding genes. Two distinct *eps* clusters are also present in *P. chungangensis* DSM_22330 and *L. nasutitermitis* CGMCC_1.15204, although the clusters appear to be incomplete or truncated in each case. In the scaffolds, the loci were manually inspected to establish if they are likely complete/intact loci or if they are fragmented/incomplete remnants. Among the 186 identified loci, 126 loci were considered intact and were identified in the genomes of strains across 14 species of the *Lactococcus–Pseudolactococcus* taxon. Currently, it is not possible to infer if the remaining 60 seemingly incomplete loci are an artefact of incomplete sequence data in the scaffolds/misassemblies or if they are remnants of ancestral loci. In addition to the 10 species for which the reference genomes were observed to possess *eps* loci, strains of *L. petauri, P. raffinolactis, P. piscium* and *P. yaeyamensis* were also observed to harbour *eps* loci. This supports the finding of the strain-specific and variable presence of *eps* loci in this taxon. Comparative analysis (30% blastP identity) further revealed extensive variability among these loci with 102 unique genetic groups and with the most abundant group containing just four representative members (Fig. 5). Consistent with this diversity, pangenome clustering of predicted *eps*-associated GT/RT proteins identified 90 homologous clusters at 30% blastP identity, increasing to 156 clusters at 70% identity. Interspecies sharing of *eps* loci was shown to be rare, with only four instances of shared clusters (allowing ≤1 divergent gene): three shared between *L. lactis* and *L. cremoris*, and one across genera between *L. lactis* and *P. chungangensis*.

**Fig. 5.**
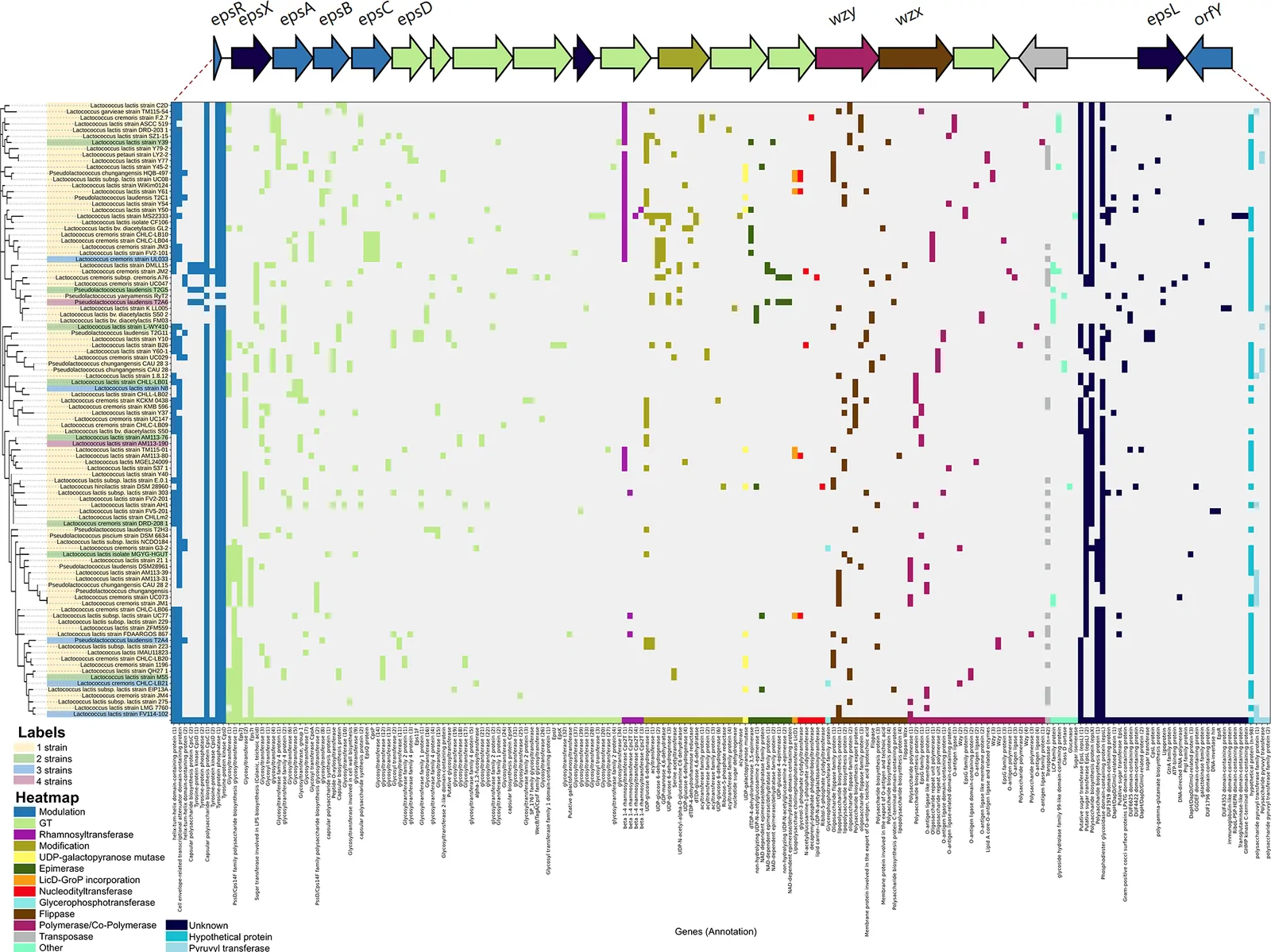
Gene presence/absence matrix for the *eps* loci across *Lactococcus* and *Pseudolactococcus* strains. Genes are grouped by predicted functional category. Presence/absence was determined from pangenome clustering using a blastP aa identity threshold of 30% to define homologous genes, accommodating substantial sequence divergence. The phylogeny is collapsed to representatives with distinct gene content profiles, with numbers in parentheses indicating how many strains each representative summarizes. The schematic at the top corresponds to the *eps* cluster of *P. laudensis* T2G11 and is provided as a reference for module content; total gene composition and synteny are not implied to be conserved across strains. Gene annotations are from Bakta, while annotations in parentheses correspond to published lactococcal annotations [25, 68]. Transposases and hypothetical proteins were collapsed to improve visualization.

Previous reports of *eps* loci among dairy lactococci have largely been associated with plasmids [66, 67, 69–71], while there are very limited reports of chromosomally harboured *eps* loci [31]. Therefore, we attempted to establish the location of the 85 *eps* loci derived from the complete genomes and for which more complete information is available pertaining to plasmid and chromosome identification. Among the *eps* loci identified in the complete genomes, 54 were associated with *L. lactis/cremoris*, whereas 31 were associated with pseudolactococcal species (*P. laudensis n*=20; *P. raffinolactis n*=5; *P. piscium, P. paracarnosus, P. carnosus n*=1) and non-dairy species of *Lactococcus* (*L. garvieae n*=2; *L. protaetieae, L. formosensis, L. petauri, L. allomyrinae n*=1). This analysis established that 31 of the 54 *L*. *lactis/cremoris eps* loci were chromosomally associated and 23 are plasmid associated. Among the 31 pseudolactococcal and non-dairy lactococcal strains' genomes that were observed to harbour *eps* loci, 30 were located in the chromosome. The genome annotations of the strains harbouring chromosomally located *eps* loci were scrutinized to investigate their genetic context. It was observed that the majority of these loci are flanked by integrase- and/or transposase-encoding genes, suggesting that these loci have been acquired by HGT. While these observations suggest a preference for a chromosomal location of *eps* loci in pseudolactococcal genomes analysed in this study, it is important to highlight the significant bias in our dataset towards the *L. lactis* and *L. cremoris,* with many of the pseudolactococcal species having low numbers of genome sequences available. Therefore, it should be noted that this observation may not reflect the complete picture of the genetic context of these loci in the lesser studied species.

To further characterize these regions, we analysed their surrounding sequences using IslandViewer4, mobileOG-db, OriTfinder2 and ICEfinder, which led to the conclusion that most chromosomal *eps* loci are embedded within predicted integrative elements. In *L. lactis* and *L. cremoris*, *eps* loci were most often associated with integrative elements or integrative and mobilizable elements (IEs/IMEs) situated upstream of the *cwps* locus. In pseudolactococci, *eps* loci were also found in predicted IEs/IMEs, but also commonly embedded within identified ICEs. In *P. laudensis*, these elements were shown to be typically located upstream of *cwps*, whereas in other analysed species their chromosomal location was more variable. Lastly, for strains in which hallmark genes of mobile elements were not detected, AlienHunter nevertheless identified the *eps*-containing regions as of HGT origin. Altogether, these observations suggest that *eps* clusters are not native features of *Lactococcus* or *Pseudolactococcus*, but these have been acquired via mobile genetic elements such as plasmids or chromosomal integrative elements in dairy *Lactococcus*, and predominantly via these integrative elements in other *Lactococcus* and *Pseudolactococcus*.

### Prophages, phage satellites and anti-phage systems in the *Lactococcus–Pseudolactococcus* taxon

CWPS represent the receptors for a number of dairy lactococcal phage genera. Given the omnipresence of *cwps* loci among lactococci and pseudolactococci, it is possible that phages of non-dairy lactococcal species may also recognize and bind to saccharidic receptors. Diversification of these CWPS structures and tuning of the expression of the associated genes are mechanisms employed by dairy lactococci to evade phages, at least temporarily [9, 72]. However, phage pressure in various ecological niches may also require bacteria to establish defences against phages to ensure their co-existence and survival in addition to the external barriers imposed by the CWPS. Therefore, we evaluated the 238 complete genomes of lactococci and pseudolactococci for the presence of prophages and phage satellites. The analysis was confined to the complete genome sequences since scaffold data may not facilitate accurate predictions of prophages as they do not have readily discernible boundaries. Additionally, for the analysis of anti-phage systems, the full dataset of complete genomes and scaffolds (*n*=495) was applied to provide an overview of the defence strategies employed by members of the *Lactococcus–Pseudolactococcus* taxon notwithstanding the obvious bias in the available data towards the dairy lactococcal species.

#### Prophage diversity highlights genetic malleability between species and genera

Using Genomad, strains for which complete genome sequences were available were scanned for the presence of potential prophages. CheckV analysis was applied to establish those of high- (>90% completeness) and medium-quality prophage regions (>80% completeness) for the purposes of this study. In this manner, from 238 complete genome sequences representing 13 species, we identified 621 prophage regions. Manual inspection of the annotated files revealed 121 prophage regions that possessed no obvious capsid or tail-associated functions and were considered as likely incomplete prophage regions. Among the genomes of 13 species (for which complete genomes were available), 11 species were observed to harbour at least one prophage region (including both predicted complete and incomplete prophages) with the average number of prophages per genome within a species ranging from 0.5 to 4 and with average genome sizes ranging from 30.7 (*L. cremoris*) to 114.6 kb (*L. allomyrinae*) (Table 1). *L. lactis* genomes were observed to harbour the highest average number of prophages per strain among all prophage-carrying species. All assessed lactococcal species (*n*=8) were observed to possess at least one prophage, while three of five pseudolactococcal species harboured identifiable prophage regions. Additional genome sequence data for all species is required to establish if there are species- or genus-specific trends in terms of prophage carriage. However, taking the example of dairy-associated species including *L. lactis, L. cremoris, P. laudensis* and *P. raffinolactis*, which are relatively well represented in this analysis, it is clear that prophages are a typical feature of their genomes, underpinning the need for diverse phage defence systems. Within species, there appears to be a range of possible regions across genomes where prophages are preferentially located (presumably dictated by their attachment or *att* sequences). Using the examples of the well-populated species *L. cremoris, L. lactis and L. petauri,* there are two to three genomic regions where prophages are most frequently observed. For example, within the genomes of *L. cremoris* and *L. lactis* strains, the regions between 1,300,000–1,500,000 and 2,000,000–2,300,000 bp appear to be particular hotspots for prophage presence, whereas in *L. petauri*, the main hotspots appeared to be 700,000 and 1,100,000–1,200,000 bp. There does not appear to be a correlation between the location of prophages in the genomes of lactococcal/pseudolactococcal strains and their *cwps/eps* loci.

**Table 1.** Summary of prophage regions haboured by lactococci and pseudolactococci

Organism	# complete genomes analysed (# genomes harbouring prophages)	# complete prophage region	# incomplete prophage region	Average # prophages per strain of species	Average genome length (kb)
*L. allomyrinae*	1 (1)	1	0	1	114.6
*L. cremoris*	41 (35)	71	38	3	30.7
*L. formosensis*	10 (6)	5	4	1.5	28.2
*L. garvieae*	25 (23)	39	0	1.7	37.1
*L. lactis*	96 (93)	321	51	4	31.4
*L. petauri*	23 (21)	28	6	1.6	31.9
*L. protaetiae*	1 (1)	2	0	0.5	33.3
*L. taiwanensis*	2 (2)	3	0	1.5	45.1
*P. carnosus*	1 (0)	0	0	0	0
*P. laudensis*	21 (18)	15	19	1.6	24.3
*P. paracarnosus*	1 (0)	0	0	0	0
*P. piscium*	1 (1)	2	0	0.5	42.9
*P. raffinolactis*	8 (8)	12	2	1.75	36.8

Comparative sequence analysis of the identified 621 prophage regions highlighted that the prophage sequences do not follow a strict genus-specific phylogeny (Fig. 6a). Indeed, the prophage regions that originate from *P. raffinolactis, P. laudensis* and *P. piscium* cluster into five phylogenetic groups that are not confined within a discrete cluster from those derived from lactococcal species. Within these five pseudolactococcal phylogenetic groups, one is predominated by *P. laudensis* prophages and with two *P. raffinolactis* prophages; one is dominated by *P. raffinolactis* prophages and with a *P. piscium* and three *P. laudensis* prophages while a third group includes primarily *P. laudensis* and one *P. piscium* prophages. The lactococcal prophage regions equally do not form discrete species-specific groups, although certain *L. formosensis, L. garvieae* and *L. petauri* phages do appear to form clusters within the overall phylogenetic tree (Fig. 6). Similarly, there is significant clustering of *L. lactis* and *L. cremoris* prophage regions, which is likely related to the ecological niche that strains of these species have shared in addition to certain strains of *P. raffinolactis* and *P. laudensis*. The clustering of prophages originating from species including *L. formosensis, L. garvieae* and *L. petauri* mirrors the finding of highly related *cwps*-associated PSP modules (Fig. 3b and c) and may allude to significant genetic exchange between these (and other) organisms and/or overlapping evolutionary pathways. However, while certain prophage clusters are consistent with strains of species sharing certain PSP-encoding *cwps* gene modules, experimental validation of the binding capabilities of these (pro)phages would be required.

**Fig. 6.**
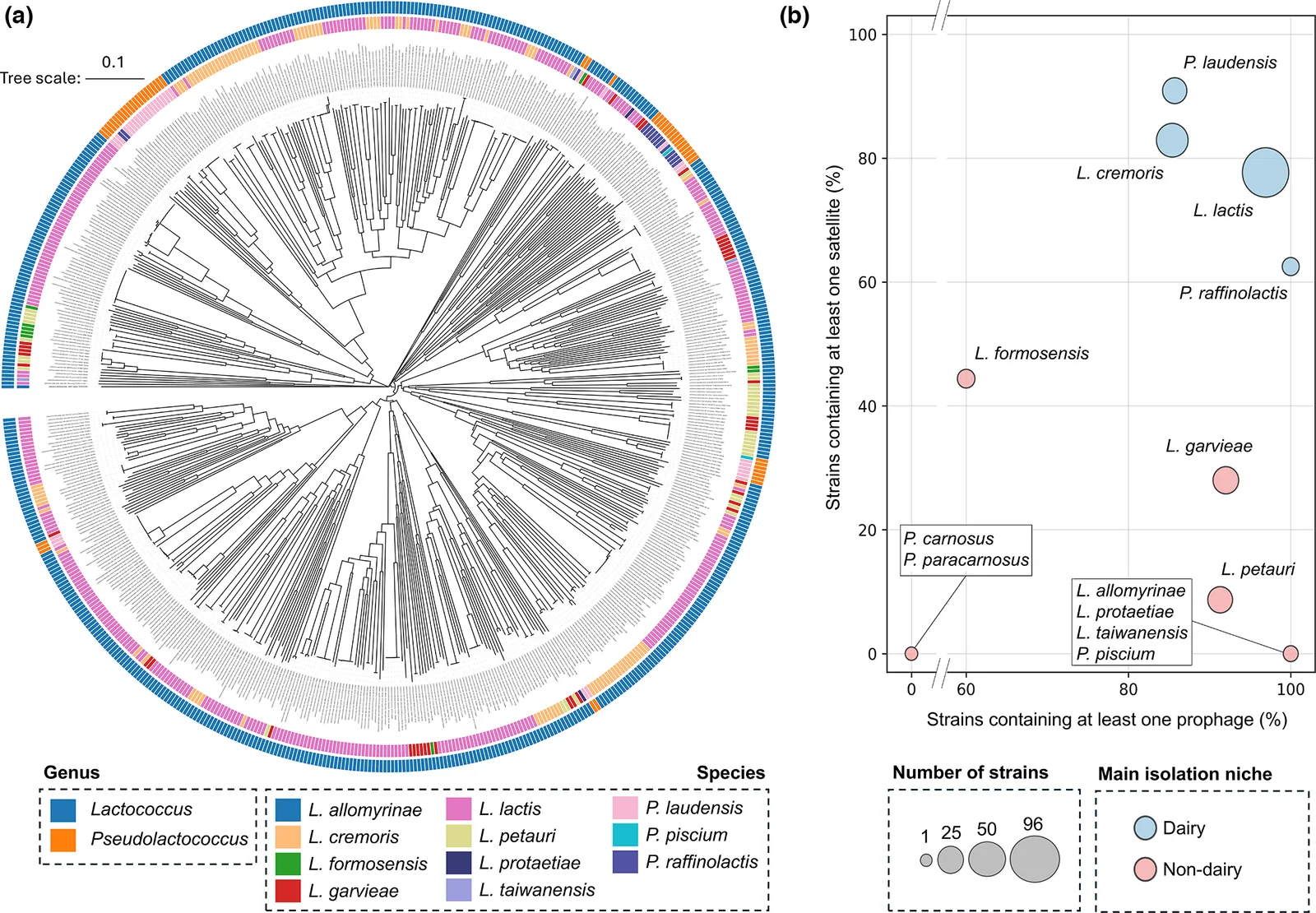
Phylogenetic diversity of prophages and prevalence of phage satellites across complete genomes of *Lactococcus* and *Pseudolactococcus*. (**a**) Unrooted phylogenetic tree of all (intact and incomplete) predicted prophage regions. The outer and inner annotation rings indicate the genus- and species-level host of each prophage, respectively, according to the colour keys below the tree. (**b**) Relationship between prophage and phage-satellite prevalence at species-level. Bubble area is proportional to the number of genomes analysed, and colour denotes if the primary ecological niche from which the species have been isolated is dairy or not. Species with identical coordinates are represented by a single bubble with their sample size combined.

#### Phage satellites are enriched in dairy-associated species

Given their dependence on helper phages, we examined the distribution of phage satellites across the same 238 complete genomes used for prophage analysis using SatelliteFinder. After discounting hits mapping to actual prophages, a total of 271 phage satellites were identified, comprising 262 PICIs (96.7%) and 9 capsid-forming cfPICIs (3.3%). Satellite prevalence varied markedly among species, ranging from 0 to 90.9%, but showed no apparent association with prophage prevalence or genus-level classification (Fig. 6b). In contrast, satellites were substantially enriched among dairy-associated species, namely *L. lactis, L. cremoris, P. laudensis* and *P. raffinolactis*, occurring in 132 of 165 genomes (80.0%), compared with 13 of 66 genomes (19.7%) from non-dairy-associated species. At the strain level, satellite abundance was positively associated with the number of intact prophages (Spearman’s ρ=0.414, p<0.001), indicating that strains harbouring more prophages also tended to carry more phage satellites. Together, these findings indicate that phage-satellite presence is associated with ecological niche, while satellite abundance is positively correlated with prophage abundance.

#### Anti-phage systems

Based on PADLOC analysis outputs, restriction–modification (R–M) systems were the most frequently identified anti-phage system in both lactococci and pseudolactococci and particularly Type I R–M systems (Fig. 7a). Additionally, certain Abi systems were frequently identified in both genera including AbiD and AbiB (Fig. 7a). A notable observation is the diverse array of anti-phage systems that are present across these taxa, likely reflecting the species- and strain-specific phage–host interactions in various niches. To define the anti-phage strategies employed at species level, a heatmap of the systems present in the genomes of strains of each species (as a percentage of the overall number of strains of a given species) was generated (Fig. 7b). Through this approach, perhaps unsurprisingly, it was evident that the highly represented species (e.g. *L. lactis, L. cremoris, L. garvieae*) possess a more diverse repertoire of anti-phage systems compared to those species for which fewer genome sequences were available. Nonetheless, these data highlight that certain anti-phage systems are present across members of both genera, while others appear to be more highly represented in the genomes of *Lactococcus* or *Pseudolactococcus* species. For example, AbiD is observed in the genomes of several species of both genera (Fig. 7b).

**Fig. 7.**
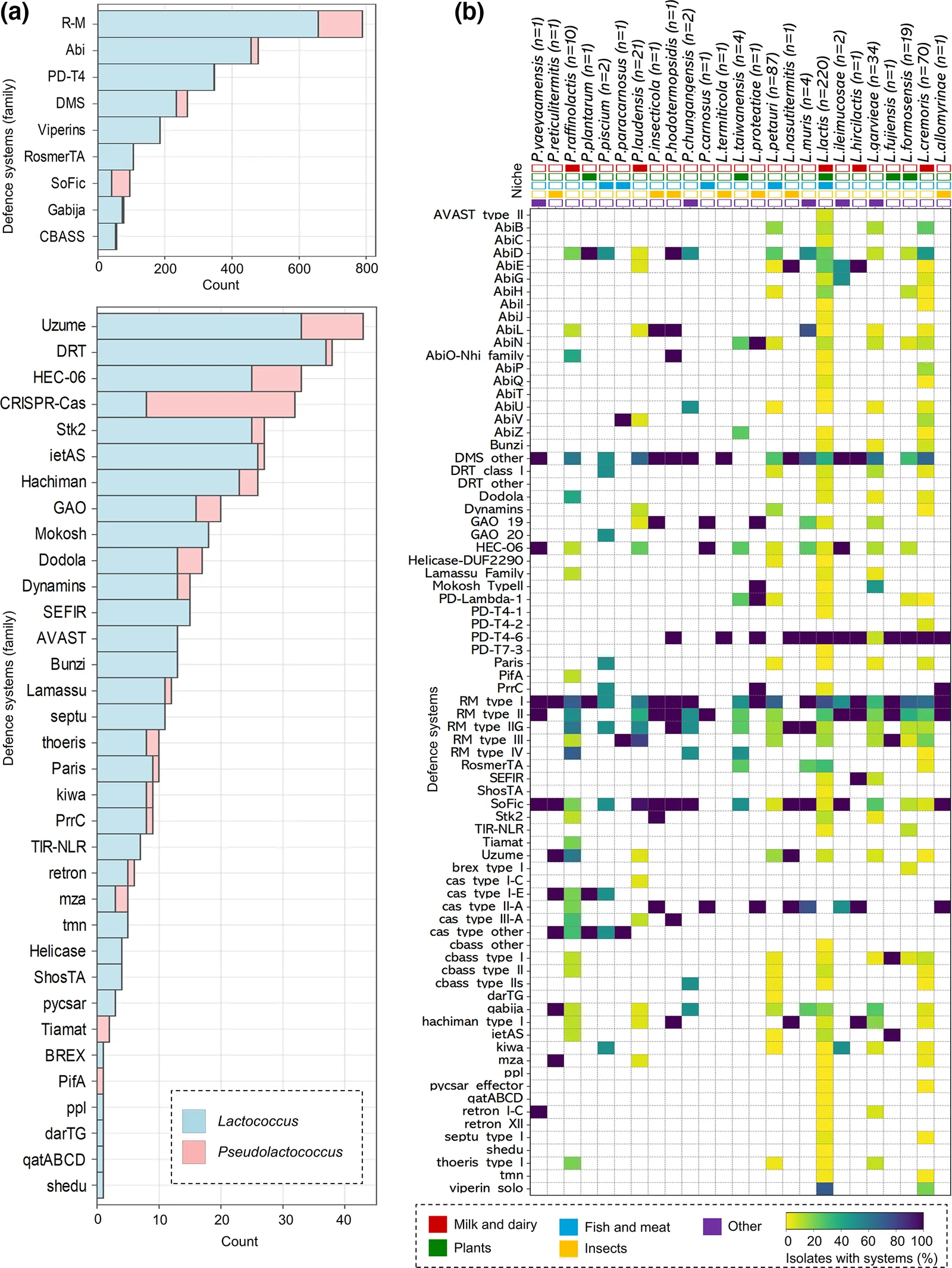
Antiphage defence systems in the *Pseudolactococcus–Lactococcus* clade detected by PADLOC. (**a**) Genus-level distribution of defence systems (summed counts across isolates), with systems grouped by family. To improve visualization, families are shown in two panels: totals≥50 (top) and totals<50 (bottom). Bars are stacked by genus. (**b**) Species-level prevalence heatmap of defence systems. Values indicate the percentage of isolates within each species carrying a given system (presence/absence only, copy number not considered). Numbers in parentheses indicate the total number of isolates analysed per species (n). Species niche associations are shown as coloured boxes above the heatmap.

There do not appear to be discernible niche-specific anti-phage repertoires based on the analysis performed here. Nevertheless, among species represented by ≥10 strains, the typically dairy-associated species *L. lactis*, *L. cremoris*, *P. laudensis* and *P. raffinolactis* carried more predicted defence systems per strain than the non-dairy-associated *L. petauri*, *L. garvieae* and *L. formosensis* (6.11 vs 2.81 systems per strain on average, respectively, for each group). This trend was also evident within the strains of *L. lactis* in which isolation metadata was available: milk/dairy isolates (*n*=40) carried more defence systems than plant-associated isolates (*n*=20) (6.33 vs 4.65 systems per strain on average). Although some species represented by a single genome also exhibited high defence-system counts (e.g. *Pseudolactococcus yaeyamensis* RyT2 contained 13 systems), these were not interpreted comparatively because of their limited representation. Future sequencing of currently underrepresented species and/or niches will be required to determine the extent to which these patterns reflect ecological adaptation rather than biases in the available genome dataset. Finally, defence-system abundance was positively correlated with both intact prophage abundance (Spearman’s ρ=0.226, *P*<0.001) and, more strongly, with satellite abundance (Spearman’s ρ=0.391, *P*<0.001). The latter is in agreement with the frequent carriage of defence-system cargo by phage satellites [73], which may contribute directly to the expanded defence repertoires of satellite-rich strains.

Finally, a thorough analysis of the CRISPR-Cas systems identified only 16 CRISPR arrays across the 238 genomes analysed, distributed among 12 strains. Of these, 11 arrays were associated with intact CRISPR-Cas systems, which were present in 10 of the 12 strains, all chromosomally encoded. CRISPR-Cas type IE, IC, IIA and IIIA are found in the genomes of pseudolactococcal species (*P. laudensis, P. raffinolactis, P. carnosus* and *P. paracarnosus*), while only type IIA is found in representatives of lactococcal *L. allomyrinae* and *L. protaetiae*, neither of which is of dairy origin (Fig. 7b). Interestingly, the previously reported plasmid-encoded type III-A system in *L. lactis* DGCC7167 [74] (not included in our dataset due to a lack of genome sequence data), shares an identical repeat with three *P. raffinolactis* (APC3967, Lr_19_5, Lr_19_14) and two *P. laudensis* (T2G5, T2H4) strains, four of which have multiple ISs disrupting the loci (except in *P. raffinolactis* APC3967) [35], raising questions about the activity of those systems (Table S2.1). The blast search of spacers against the 238-genome dataset showed that across all 12 CRISPR-carrying strains, the total of 77 spacers returned hits and that they predominantly targeted prophages (476 hits), with 4 *P*. *raffinolactis* Lr_19_5 spacers targeting plasmids (11 hits), but no hits to phage satellites were found. Various hits crossed the host species barrier and in case of pseudolactococci (other than *P. laudensis*) crossing the genus barrier as well. Interestingly, the type III-A spacers of the prophage-free *P. laudensis* strains T2G5 and T2H4 matched an intact prophage present in five other *P. laudensis* strains isolated from the same starter culture (T2A7, T2C9, T2D8, T2E11 and T2H3) [35], consistent with active CRISPR exclusion of a prophage that lysogenized the co-cultured strains (Table S2.2).

The additional blast search against 466-genome lactococcal phage database (INPHARED) resulted in only 26 spacers returning hits, of which only 4 and none matched against defined lytic Skunaviruses and Ceduoviruses*,* respectively. Of the 26 spacers, only 8 did not also match resident prophages from the analysed genomes, including 3 of the 4 spacers matching Skunaviruses. These results support the notion that acquisition of spacers against lytic phages is less frequent due to the quick cell death but also imply that some of the spacers may have been acquired during a temperate rather than lytic phage infection [75].

It is also worth noting that 20 of the 26 spacers returning hits originated from *Pseudolactococcus* CRISPR arrays (*P. raffinolactis, P. paracarnosus* and *P. carnosus*) and 17 of those spacers matched 179 phage genomes whose recorded isolation host is dairy-associated *L. lactis* or *L. cremoris*, including perfect-identity matches, potentially pointing to a broader host range across the *Lactococcus/Pseudolactococcus* boundary than historically assumed, although this would require experimental validation. By contrast, spacers from dairy *P. laudensis* strains matched no external phages, consistent with their CRISPR memory being dominated by species-specific elements rather than the broadly distributed phage population (Table S2.3).

## Discussion

Dairy lactococcal species and their infecting phages have been extensively studied over the past four decades resulting in a wealth of information on their biology and interactions [6, 8, 57, 62–66, 76–79]. Ultimately, the derived information is essential in guiding the selection of strains for application in defined starter cultures and in understanding the population dynamics in mixed strain starter culture systems [80, 81]. Furthermore, dairy lactococcal species have been widely reported to harbour an array of anti-phage systems, primarily represented by R–M systems and with a variety of Abi systems [33, 82–85]. Indeed, *L. lactis* and *L. cremoris* have been model organisms for the identification and characterization of Abi systems [86]. In contrast to other dairy-associated species including *S. thermophilus,* where CRISPR-Cas systems are prevalent and a primary anti-phage strategy, there have been very limited reports of CRISPR-Cas systems in lactococcal species [34, 87].

The recent expansion of the lactococcal genus and the reclassification of certain species to the *Pseudolactococcus* genus has revealed gaps in our understanding of the genetics and biology of the *Lactococcus–Pseudolactococcus* taxon. For example, while in dairy lactococci, *cwps* loci diversification is believed to be attributable (in part, at least) to persistent phage pressure in the fermentation environment, it is unclear if there is similar diversity in these loci in other *Lactococcus* and *Pseudolactococcus* species, and if this may be a tool to predict if similar phage pressure exists among them. The presence of prophages is well defined in dairy species but is less well studied in non-dairy species. Finally, while these individual features have been explored in detail in separate studies, there are very few reports within any species that provide an overview of all the phage-relevant features in their genomes. Therefore, in the present study, we sought to provide a holistic overview of the presence and diversity of cell surface polysaccharide related loci as potential phage receptors across all species (including CWPS and EPS). Secondly, we aimed to define the presence of prophages in the available complete genomes as a predictor of phage pressure that requires the presence and activity of anti-phage systems. Among lactococcal/pseudolactococcal species for which 10 or more genome sequences are available, it appears that a higher number of genomes is directly correlated with a similarly increasing number or types of anti-phage systems while the number of predicted prophages per genome does not increase. However, in species such as *L. lactis, L. cremoris, L. petauri, P. laudensis* and *P. raffinolactis,* the diversity of phages (based on phylogenetic analysis) increases. From this, we infer that the diversity of phages within a given species, rather than the number of prophage per genome, is an indicator of the phage pressure and may be linked to an increased requirement for diverse anti-phage systems. However, this will require robust and detailed analysis as more genome sequence data becomes available for both genera and their component species. Nonetheless, we present the most comprehensive overview of phage-relevant features in the *Lactococcus–Pseudolactococcus* taxon to date.

The role of CWPS among dairy lactococci is well established [7, 72, 88]. Beyond *L. lactis* and *L. cremoris*, we have now established that this locus is universally present in a similar genomic position (relative to *dnaA*) in all species within the *Lactococcus–Pseudolactococcus* taxon (Fig. 1). These loci are highly diverse across the taxon indicating that regular phage encounters are likely a feature of all species. Additionally, it is noteworthy that there is significant inter-species *cwps* locus overlap indicating the modularity of these gene clusters, resulting either from a shared common ancestor or their ready transfer between species through homologous recombination. Since the *cwps* locus is a universal feature of all members of the taxon and there are no clear signs of HGT, it seems most likely that these species are derived from a common ancestor. The location of the *cwps* locus relative to the origin of replication signifies its importance as these genes will be replicated early in the cell cycle and are therefore typically present in higher copy number than genes located toward the replication terminus [89]. In contrast, *eps* loci are a variably present feature in several species of the taxon that may be located on the chromosomes or plasmids of given strains of the species that harbour them. The *eps* loci were typically observed to be associated with predicted IEs or IMEs, indicating that they are acquired by strains probably to boost their niche adaptation, biofilm formation and likely persistence in various environmental contexts. However, to fully validate this hypothesis, higher resolution analysis of strains of the *eps* locus carrying species and matching metadata relating to their source niche would be required. In the context of phage–host interactions, EPS can mask cell surface receptors for particular phages, although they have also been reported to mediate binding of certain lactococcal phages [8, 28]. Therefore, the variable presence of these loci in species of this taxon highlights their genomic plasticity and potential HGT between distantly related species. The finding of eight strains lacking core functions associated with PSP synthesis and transfer to the rhamnan backbone highlights a potential adaptation to phage pressure or other external pressures. It has previously been demonstrated in *L. cremoris* that reduced synthesis of the PSP component is associated with (short-term) phage evasion [90]. While the rhamnan component is essential and cannot be compromised, the PSP is a non-essential component whose absence may be a benefit in certain circumstances. The lack of a side chain has been associated with certain growth defects such as long chain formation and reduced cell division capabilities and may explain the apparent low incidence of this genotype presentation [88].

Given the important role of surface polysaccharides in dairy fermentations, both as key determinants of phage–host interactions and, in the case of EPS, also as contributors to technologically valuable traits [27], being able to predict polysaccharide phenotypes from genomic data would be highly advantageous. Although initial genotype-to-phenotype characterizations for lactococcal and pseudolactococcal surface polysaccharides have been performed [9, 25, 26, 40], the extensive diversification of GTs observed here, together with the high prevalence of strain-specific genotypes (particularly among *eps* loci), indicates that robust structural prediction will require elevated research efforts.

The relevance of CWPS diversity may extend beyond dairy fermentations to the control of pathogenic lactococci. *L. garvieae* and *L. petauri* are important agents of piscine lactococcosis, *L. formosensis* has also been increasingly associated with disease in aquaculture, and *L. garvieae* has been implicated in bovine mastitis [91–94]. Phage-based interventions are being explored as alternatives or complements to antibiotics in aquaculture, with experimental protection demonstrated against *L. garvieae* in yellowtail, as well as against other major fish pathogens [95, 96]. The comparative framework established here may therefore facilitate the selection of genetically representative isolates for phage screening and support the development of phage combinations targeting distinct receptor types.

Following adsorption and genome injection, temperate phages may integrate into the bacterial chromosome as prophages, remaining dormant until conditions become non-conducive and trigger induction and activation of the lytic cycle. Dairy lactococci have previously been described to harbour up to six prophages, highlighting the obvious co-evolution and existence of phages with their hosts in this niche [97]. Prophage-associated holin-lysin systems have also been proven to activate extracellular vesicle production [98], potentially facilitating genetic exchange between bacterial strains and species that co-exist. In parallel, bacterial DNA can be packaged into phage capsids during their assembly and be subsequently transduced into new hosts in subsequent infections [99]. Therefore, the presence of prophages is an indicator of not only phage exposure, co-existence and possible evolutionary pressure within a species, but also of potential DNA transfer between species. Here, we established that most species for which complete genome sequence data are available harbour prophages. However, it is clear that additional sequence data will enhance our ability to understand the full extent and diversity of the (pro)phages within this taxon. Furthermore, while an abundance of prophages was identified in the analysed genomes, the functional assignment of their gene content is limited. Therefore, future efforts should focus on improving the annotations of these phages to facilitate the identification of potential adhesion-associated genes and their potential relationship to strains of a given *cwps* genotype. However, it should be noted that such interpretations would require biological validation using binding assays, for example, to accurately assign host *cwps* genotype to (pro)phage receptor binding protein-encoding genotypes since the *cwps* locus is clearly a highly variable region, which will likely evolve in the face of repeated phage exposure (among other pressures).

Phage satellites provide an additional link between phage exposure, mobile genetic elements and bacterial defence. In agreement with their dependence on helper phages, satellite abundance was positively correlated with intact prophage abundance, suggesting that prophage-rich genomes may provide greater opportunities for satellite maintenance or mobilization. The enrichment of PICIs particularly among dairy-associated species may reflect selection imposed by persistent phage exposure in dense, repeatedly propagated starter cultures. By redirecting helper-phage replication, assembly and packaging functions for their own dissemination [100], classical PICIs can substantially reduce the production of infectious phage particles and may therefore function as non-canonical, population-level defence systems widespread in dairy environments.

Beyond satellites, our analysis indicated that R–M systems are highly prevalent in the analysed genomes and likely represent the core phage defence strategy of the taxon. The R–M systems are supplemented by a species and even strain-level panel of Abi and/or DNA cleavage systems (such as CRISPR-Cas, BREX, etc). This mirrors our recent studies of dairy lactococci albeit with a broader repertoire of anti-phage systems in the current study [33]. Furthermore, with the recent surge in research into novel anti-phage systems, it is likely that many more such systems will be uncovered in the coming years, which will enhance our understanding of the anti-phage strategies applied by lactococcal and pseudolactococcal species following functional validation of their activity [101–104]. The finding of seemingly shared anti-phage systems suggests that there are shared phage sequences and/or proteins that may be recognized by DNA cleavage systems or may trigger or be targeted by Abi systems, respectively. Analysis of eight predicted intact prophages of *L. garvieae* demonstrated similarity in sequence and G+C mol% content to *L. lactis* P335 temperate phages [105]. In the future, detailed comparative genomic analysis of the prophages of species within this taxon will be performed to fully understand the relationship between them and their hosts.

Since strains of several lactococcal and pseudolactococcal species are isolated from different ecological niches, it seems likely that many of these have a broad capacity for growth under an array of environmental conditions creating opportunities for genetic exchange. In contrast, strains of *L. lactis/cremoris* serve as examples of exquisite niche adaptation and genome decay and reduction [106], which may yet be discovered in more recently emerging species. The genome decay reported in these dairy species has been linked to a relatively high number of transposons and pseudogenes while the niche adaptation was attributed based on a large plasmid complement. Despite the apparent genome decay, the diversity of *cwps* genotypes among strains of these species highlights the genomic elasticity within these loci that further enhance their evolutionary capacity. The universal presence and conserved genomic position of cwps across the *Lactococcus–Pseudolactococcus* clade, and the observation of shared gene content from these loci in diverse species, support inheritance from a common ancestor, with related PSP loci across species and niches possibly reflecting differential retention and diversification of ancestral variants. Their current distribution may therefore have been shaped by ecological selection and expansion of successful lineages. Consistent with this model, dairy-associated *L. lactis* strains carried more prophages, satellites and defence systems than their plant-associated counterparts, yet exhibited lower rarefied PSP richness. Repeated industrial propagation of successful dairy lineages may have narrowed the range of PSP configurations represented in this niche, while favouring the accumulation of intracellular and satellite-associated defences. In contrast, the greater ecological heterogeneity of plant environments may help maintain a broader catalogue of ancestral PSP diversity. Finally, we note that disproportionate sampling of closely related dairy strains can also contribute to this pattern.

In conclusion, we have established the universal presence of *cwps* loci across the *Lactococcus–Pseudolactococcus* taxon and with approximately half of the analysed species genomes harbouring *eps* loci. This highlights the integral nature of CWPS across the taxon in the biological functions of these organisms while EPS may represent a niche adaptation that has been acquired by certain species. Furthermore, the observed phylogenetic overlap of prophages across several species of both genera may highlight shared ancestral origins or shared phage–host interaction types across these species. As genome sequencing continues to become a routine activity in global research laboratories, it is highly likely that the *Lactococcus–Pseudolactococcus* taxon will continue to expand far beyond the dairy niche. Here, we have generated insights into the shared and unique genetic attributes of species within the taxon and phage-related features of their genomes. It also deepens our understanding of the true omnipresence of species of *Lactococcus* and *Pseudolactococcus* in diverse ecological niches being now more consistent with other ovococcoid lactic acid bacteria, including the streptococci and enterococci [107, 108]. This will facilitate more comprehensive inter-genus comparisons within the ovococcoid lactic acid bacteria to understand the evolutionary pathways of these fascinating organisms.

## Supplementary material

10.1099/mgen.0.001863 Supplementary Material 1.

10.1099/mgen.0.001863 Supplementary Material 2.

## Abbreviations

Abi: abortive infection
CDS: coding sequence
CWPS: cell wall polysaccharides
EPS: exopolysaccharides
GT: glycosyltransferase
HGT: horizontal gene transfer
ICE: integrative and conjugative element
IE: integrative element
IME: integrative mobilizable element
IS: insertion sequence
PICI: phage-inducible chromosomal island
PSP: polysaccharide pellicle
R–M: restriction–modification
RT: rhamnosyltransferase
