# Supplementary material for: Members of the Lactococcus–Pseudolactococcus clade exhibit extensive genomic diversity in features associated with phage–host co-adaptation
Source: Microb Genom. 2026 Oct 12;12(10):001863. doi: 10.1099/mgen.0.001863 (PMC13657430; doi:10.1099/mgen.0.001863)
Supplement: Supplementary file 1 [file mgen-12-01863-s001.pdf]

Table S1. Lactococcal and pseudolactococcal reference strains and genome information

Species	Isolation source	# of complete genomes/scaffolds available	Average genome size (Mb)	NCBI Genome accession number and strain analysed in this study	# contigs for reference strain	Reference
L. allomyrinae	Gut of *Allomyrina dichotoma* larva	1/1	2.8	ASM362709v1<br>1JSPR-7	3	[12]
L. taiwanensis	Fresh cummingcordia	2/2	1.9	ASM1706835v1<br>K_LL004	1	[21]
L. termiticola	Gut of *Nasutitermes takasagoensis*	0/1	1.7	ASM311683v1<br>NtB2	22	[16]
L. cremoris	Dairy	41/29	2.5	ASM46895v1<br>KW2	1	[29]
L. formosensis	Fermented broccoli stems	10/9	2.2	ASM3789237v1<br>M0402041EE5e5	1	[14]
L. fujiensis	Chinese cabbage leaves	0/1	2.1	ASM244165v1<br>JCM16395	69	[30]
L. garvieae	Bovine mastitis, fish pathogen	25/12	2.1	ASM1602669v1<br>FDAARGOS_929	2	[31]
L. hircilactis	Goat milk	0/2	2.6	ASM960101v1<br>DSM28960	50	[15]
L. protaetiae	Gut of *Protaetia brevitarsis seulensis* larva	1/0	2.7	SM696544v1<br>KACC19320	1	[12]
L. ileimucosae	Jejunal/ileal mucosa of a wild mouse	0/2	2.2	ASM4122548v1<br>26-13	46	[32]

L. petaurid	Facial abscess of the sugar glider	23/64	2.1	ASM2349927v1 B1726	1	[33]
L. kimchi	Commercial baechu-kimchi in Korea	0/0	NA	NA	NA	[34]
L. lactis	Dairy, plant	96/124	2.5	ASM317683v1 14B4	2	[35]
L. muris	Caecal content of a mouse	0/4	2.65	ASM4284911v1 M2458	51	[32]
L. nasutitermitis	Gut of *Nasutitermes hainanensis*	0/1	2.2	ASM1840376v1 NBRC111537	12	[18]
P. paracarnosus	Red meat	1/0	2.2	ASM677028v1 TMW2.1615	1	[11]
P. laudensis	Goat milk	21/1	2.3	ASM1344973v1 DSM28961	69	[15]
P. piscium	Salmonid fish	1/1	2.3	ASM244169v1 DSM6634	75	[36]
P. plantarum	Frozen peas	0/1	2.0	ASM159174v1 NBRC100936	46	[31]
P. insecticola	Gut of *Hodotermopsis sjostedti*	0/1	2.0	ASM1117006v1 Hs20B0-1	28	[17]
P. raffinolactis	Milk, dairy	8/2	2.3	ASM1066924v1 KACC13441	1	[37]
P. reticulitermitis	Gut of *Reticulitermes speratus*	0/1	2.2	ASM226084v1 Rs-Y01	20	[20]

P. hodotermopsidis	Gut of *Hodotermopsis sjostedti*	0/1	2.3	ASM1117008v1	138	[17]
P. yaeyamensis	Gut of *R. yaeyamanus*	0/1	NA	BAAGGP010000001–BAAGGP010000192	192	[23]
P. carnosus	Red meat	1/0	2.2	ASM677026v1 TMW2.162	1	[11]
P. chungangensis	Activated sludge	0/4	2.1	ASM244188v1 DSM22330	87	[38]

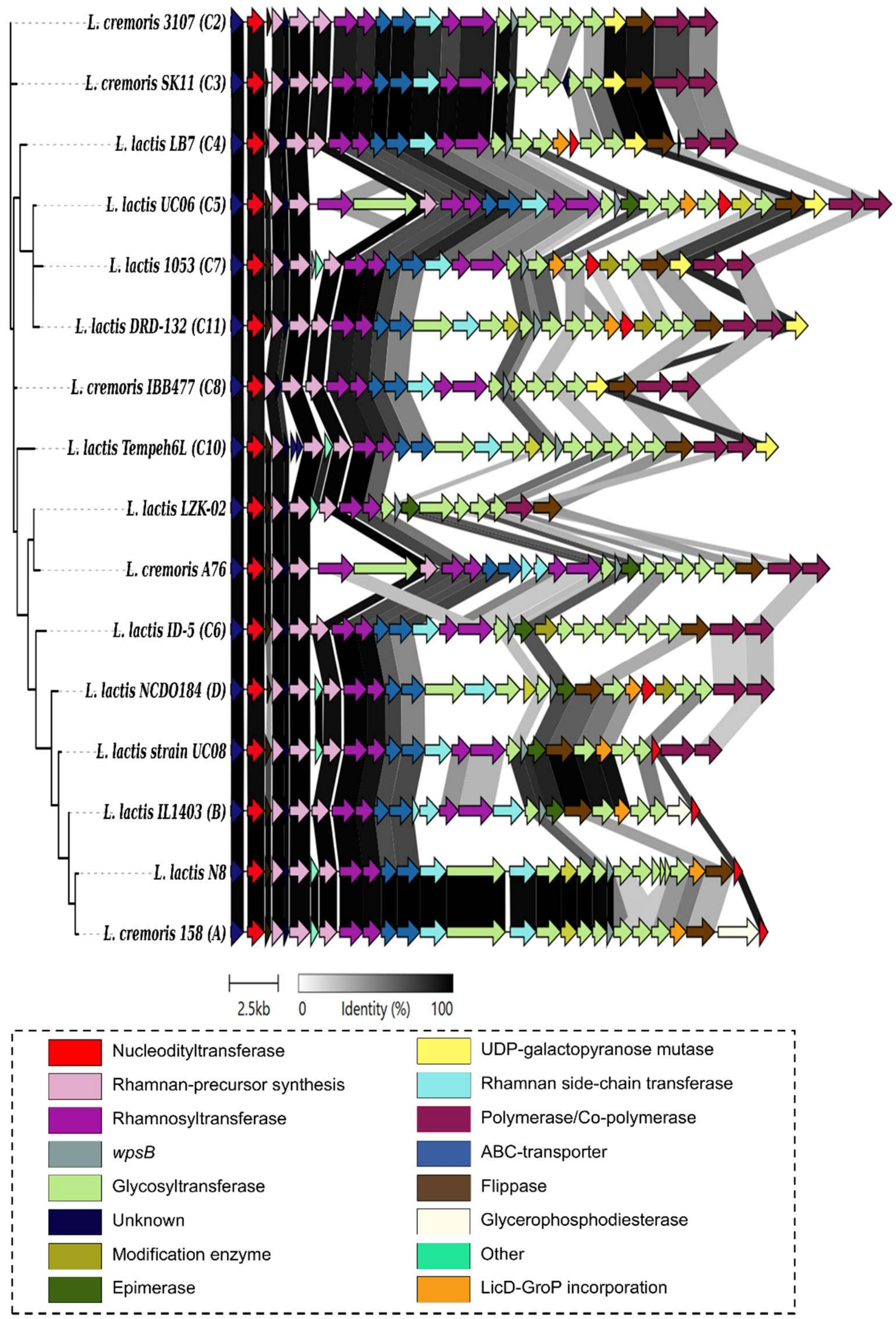

Supplementary Fig. S1. Representatives of *L. lactis* and *L. cremoris* most dissimilar genotypes based on their side-chain module. Former classification of the strains into the established A-D system is included in parentheses, when available.

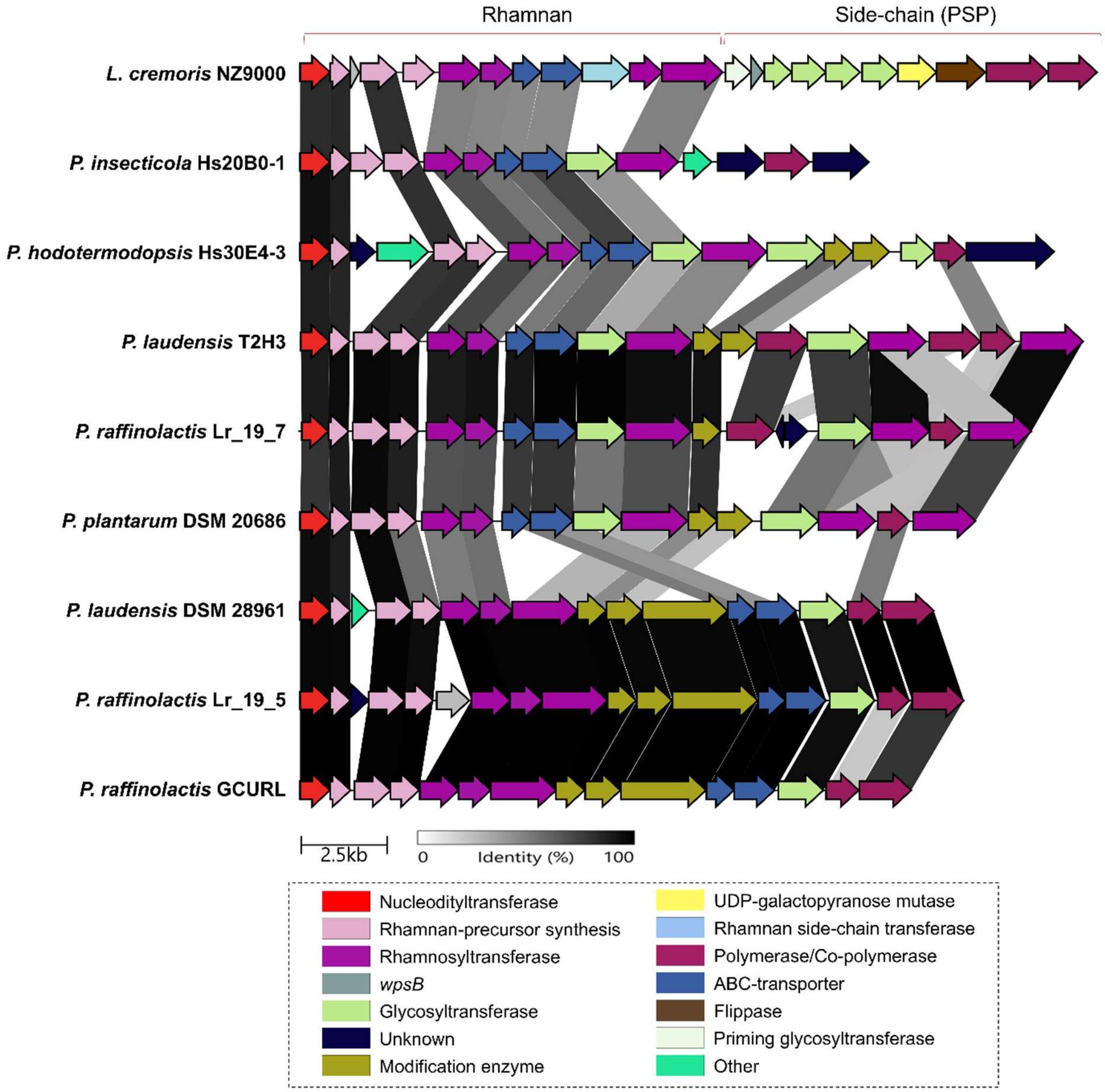

Supplementary Fig. S2. *Pseudolactococcus* strains with no PSP-encoding genes. The strains lack hallmark side-chain biosynthesis and assembly genes such as the priming glycosyltransferase *wpsA, wpsB*, and the side-chain transferase. The *cwps* cluster of *L. cremoris* NZ9000 has been added as a reference.

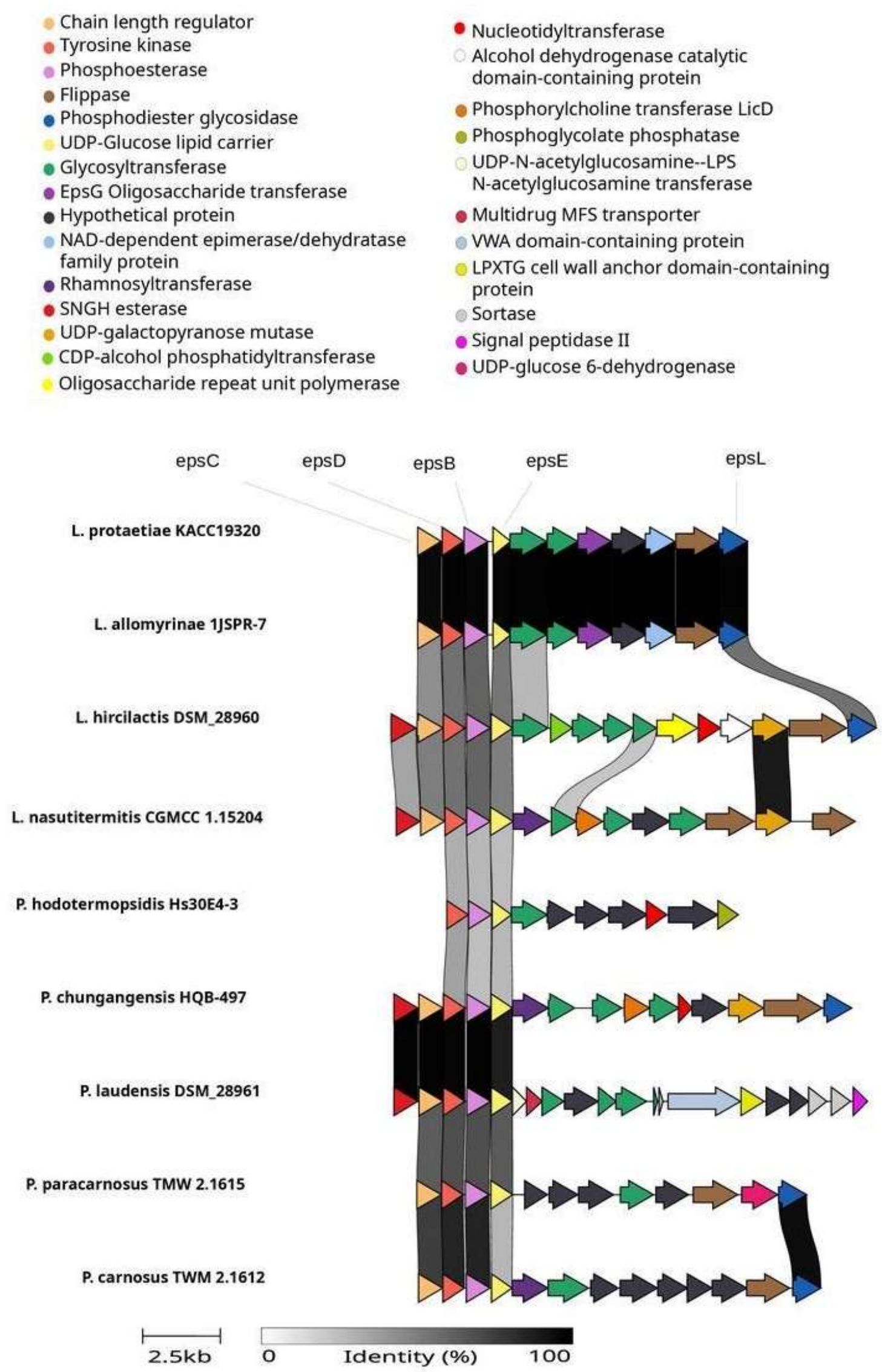

Supplementary Fig. S3. Representation of the *eps* loci identified in the reference genomes of lactococcal and pseudolactococcal species. Predicted functions are colour-coded according to the colour scheme above the figure.
